# Structural, Electronic, Elastic, and Optical Characteristics of AgZF_3_ (Z = Sb and Bi) Fluoro-Perovskites: Using a Computational Approach for Energy Generation

**DOI:** 10.3390/molecules28114418

**Published:** 2023-05-29

**Authors:** Fekhra Hedhili, Hukam Khan, Mohammad Sohail, Nasir Rahman, Rajwali Khan, Waed Alahmad, Hissah Saedoon Albaqawi, Shereen Mohammed Al-Shomar, Omar Alsalmi

**Affiliations:** 1 Department of Physics, College of Science, University of Ha’il, P.O. Box 2440, Ha’il 81451, Saudi Arabia; f.hedhili@uoh.edu.sa (F.H.); h.albuqawi@uoh.edu.sa (H.S.A.); s.alshomar@uoh.edu.sa (S.M.A.-S.); 2 Department of Physics, Faculty of Science, Al Manar University, Tunis 1060, Tunisia; 3 Department of Physics, University of Lakki Marwat, Lakki Marwat 28420, Khyber Pakhtunkhwa, Pakistan; hkmarwat80@gmail.com (H.K.); nasir@ullm.edu.pk (N.R.); rajwali@ulm.edu.pk (R.K.); 4 Department of Chemistry, Faculty of Arts and Science, Applied Science Private University, P.O. Box 166, Amman 11931, Jordan; w_alahmad@asu.edu.jo; 5 Physics Department, Faculty of Applied Science, Umm Al-Qura University, P.O. Box 715, Makkah 24382, Saudi Arabia; ohsalmi@uqu.edu.sa

**Keywords:** condense matter physics, fluoro-perovskite, visual properties, structural properties, electronic properties

## Abstract

This research is being conducted to learn more about various compounds and their potential uses in various fields such as renewable energy, electrical conductivity, the study of optoelectronic properties, the use of light-absorbing materials in photovoltaic device thin-film LEDs, and field effect transistors (FETs). AgZF3 (Z = Sb, Bi) compounds, which are simple, cubic, ternary fluoro-perovskites, are studied using the FP-LAPW and low orbital algorithm, both of which are based on DFT. Structure, elasticity and electrical and optical properties are only some of the many features that can be predicted. The TB-mBJ method is used to analyze several property types. An important finding of this study is an increase in the bulk modulus value after switching Sb to Bi as the metallic cation designated as “Z” demonstrates the stiffness characteristic of a material. The anisotropy and mechanical balance of the underexplored compounds are also revealed. Our compounds are ductile, as evidenced by the calculated Poisson ratio, Cauchy pressure, and Pugh ratio values. Both compounds exhibit indirect band gaps (X-M), with the lowest points of the conduction bands located at the evenness point X and the highest points of the valence bands located at the symmetry point M. The principal peaks in the optical spectrum can be understood in light of the observed electronic structure.

## 1. Introduction

Researchers working on perovskites are creating new substances with improved properties. The structures of substances with the chemical formula ABF_3_ are typically fluoro-perovskites. The perovskites CaTiO_3_ were the first materials that exhibit this material’s atomic arrangement. In the structure of a perovskite, there are twelve atoms of cation “A”, and six atoms of B are bound to it. Fluoro-perovskite complexes comprise a special class of ingredients that traverse from insulators to semiconductors with stable crystalline arrangements and exceptional electrical properties. These compounds have gained significant consideration lately due to their critical importance and the fact that they can be used as lens materials in photonic lithography and in radiation dosimeters as scintillating materials. The semiconductor manufacturing [1,2,3] process can recycle them as visual materials in lithography. These substances have generated a lot of discussion in recent years. Numerous studies have looked at the properties of Perovskites compounds, and the majority have come to the conclusion that they are anisotropic [4,5,6]. ABF_3_ composites have a variety of uses, including as highly efficient photovoltaic components and as excellent energy storage for automobiles and electronic and visual devices [7,8,9]. To create steady fluoro-perovskites, fluorine is mixed with inorganic or organic substances and a group of transition metals. The most practical options are broad gap fluoro-perovskites. These may be coupled to create intricately matched lattice materials with huge bands, allowing for band gap engineering [1]. Their wide energy band gap is characteristic of these molecules. Due to their high potential and weak absorption edges, these compounds can be utilized to create glass in vacuum ultraviolet and ultraviolet wavelengths [10,11]. As shown in the references, new research on fluoro-perovskites has recently been described [12,13,14]. DFT was used by Harmel in reference [15] to investigate the characteristics of barium BaCsF3 fluoro-perovskites, and they came to the conclusion that BaCsF3 is appropriate for use in opto-electronic devices due to its wide-ranging direct bands and ensembles of the unreal component of the insulating characteristics in the ultraviolet region. In their discussion of a few properties of LiBaF3, Daniel et al. [16] discovered that these chemicals are appropriate for storing energy.

In this work, a significant class of novel and valuable ternary compounds, AgZF_3_ (Z = Sb, Bi), is introduced. These complexes have the capacity to be exploited as optical materials in contemporary electrical technologies. Using the simulation software wien2k, we found that AgBiF_3_ exhibits a high level of electrical conductivity, making it a viable alternative for conduction in electrical usage. AgSbF_3_ is a superb option for electrical applications in intermediate energy ranges since it has been found to pass electrical signals with exceptional transparency over restricted energy barriers. To our knowledge, there is little literature on Ag-based fluoro-perovskites regardless of the attention paid to fluoro-perovskites and their use in a number of applications.

## 2. Results and Discussion

This part of our research work presents a detailed output and scientific discussion after the application of TB-MBJ potential methods to explore the behavior of the compounds presented herein. In this segment, we shall explain physical properties such as structural, optical, and elastic properties.

### 2.1. Structural Properties

**Figure 1:**
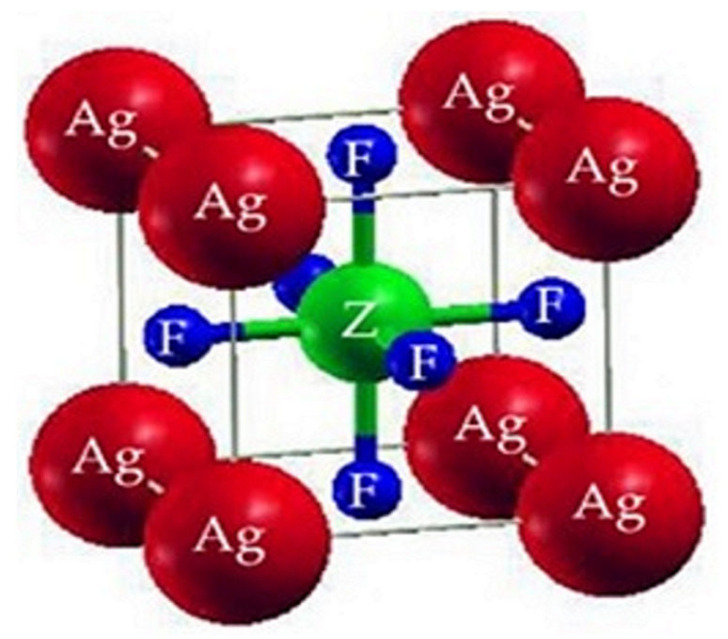
The prototypical crystal structure of ternary compound AgZF_3_ (Z = Sb, Bi).

**Figure 2:**
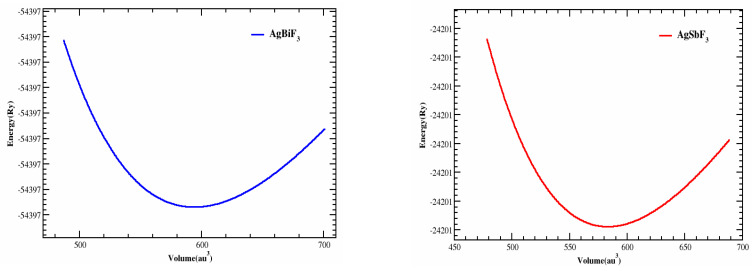
The optimized curve of AgZF_3_ (Z = Sb, Bi) complexes built in by the Birch–Murnaghan equation.

AgZF_3_ (Z = Sb, Bi) crystallizes to construct a Pm3m (#221) cubic perovskite with one composite acting as the unit cell. Z atoms (Z = Sb, Bi) are found at position (1/2,1/2,1/2), while F atoms are found at positions (0,1/2,1/2), (1/2,0,1/2), and (1/2,1/2,0). Ag atoms are found at position (0, 0, 0). Figure 1 illustrates the cubic structures of the Ag-based fluoro-perovskite compounds. The total energy was designed in terms of the unit cell volume around the Vo (the group volume at the balance condition). The optimal volume tactic can be recycled to estimate the structural limitations by utilizing the Birch–Murnaghan equivalence of states [17]. By fitting and obtaining the lowest state characteristics, such as the bulk modulus B, the variation in pressure, and the lattice constant, ao, at equilibrium, we produced logical evaluation curves, shown in Figure 2, of the cell’s bottommost energy at the proper volume. The total minimum energy versus the volume represents the ideal or ground condition, Eo. Vo is the term given to the quantity: it stands for the ideal or bottommost state minimal volume. It is anticipated that the compound with the peak optimized energy will have a more stable structure. The Ao (optimized lattice constant), Eo (optimized ground state energy), Bo (bulk modulus), Vo (optimized volume), and Bo (bulk modulus) are among the ideal structural characteristics that were discovered. Table 1 contains a list of them (derivatives of the bulk modulus vs pressure). Since B decreases as the lattice number increases, these outcomes are consistent with the general tendency of this technique, proving that the estimated values are more true and practical. AgBiF_3_ is structurally different from AgSbF_3_ based on the steeper optimization fit curve.

### 2.2. Electronic Properties

**Figure 3:**
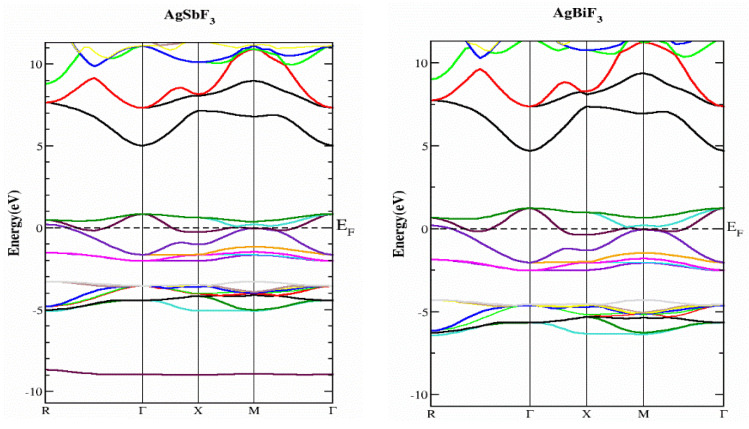
Energy band structures of compounds AgZF_3_ (Z = Sb, Bi), using the TB-mBJ approximation.

**Figure 4:**
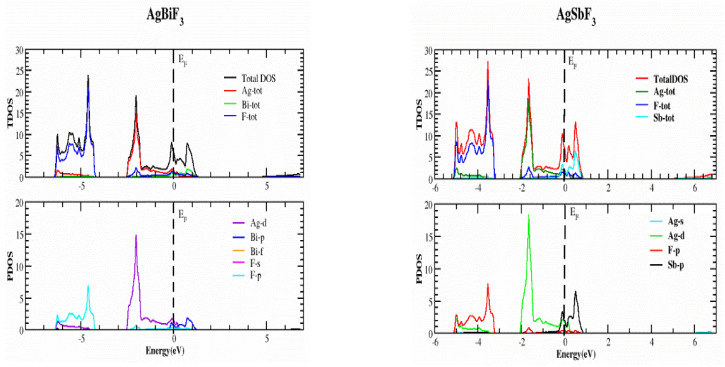
Representation of the TDOS and PDOS of the AgZF_3_ (Z = Sb, Bi) compounds, using the TB-mBJ approximation.

In this part, we study the electrical characteristics of AgZF_3_ complexes (Z = Sb, Bi) by establishing the actual band structure diagrams, DOS, and charge distribution diagrams. The band separation of semiconductors and insulating materials can be originated using well-known LDA and GGA computations [17,18]. The main reason for this is the inability of their fundamental geometries to reliably recreate the exchange-correlation technique and its change in charge. The underestimation of the band parting was resolved using the TB-MBJ approach, which has been effectively utilized in many recent publications [15,19,20]. The detected energy band structures for the AgZF_3_ geometry (Z = Sb, Bi) at equilibrium along directions of great symmetry are provided in Figure 3. The null energy level at the peak of the valence band is chosen to be the Fermi energy, EF. Both compounds are metals because their valence band (VB) peaks and conduction bands greatly overlap. Conduction group minima are positioned at balance point X, whereas lower band peaks are located at balance point M, resulting in an indirect (X-M) for AlAgF_3_. AlSF_3_, on the other hand, exhibits a metallic nature since its conduction band minima are at “X” and cross the Fermi energy level. In order to better understand the electronic assembly, the TDOS and PDOS (total and partial concentration of states) of AgZF_3_ complexes (Z = Sb, Bi) are shown in Figure 4. The vertical dashed lines at EF = 0.0 eV represent the Fermi energy region, whereas the DOS ranges from −6.0 to 6.0 eV. While the valance band is to the left of EF, the conduction band of the DOS is to the right. The F-tot, F-p, and Ag-d states are all in the valence band for AgBiF_3_, with energies varying from −4.20 to −6.40 eV and 0.0 to −2.50 eV, contributing prominently.

Bi-p, F-tot, and Bi-tot make small contributions in the conduction band in a range of energy varying from 0.0 to 1.0 eV for the same compound. For AgSbF_3_, F-tot, Ag-tot, F-p, and Ag-d make the largest contributions in on the left-hand side of the band, with the energy changing from −3.20 to −5.40 eV, from −3.8 to −5.40 eV, from −3.20 to −5.40 eV, and from −3.80 to −5.40 eV respectively. Similarly, in the conduction band of the same compound, the chief influence emanates from Sb-p, whereas the F-tot in the energy varies from 0.0 to 1.0 eV for both states, as illustrated in Figure 4.

### 2.3. Elastic Properties

The elastic factors in reaction to the exterior forces on the composite can be used to compute the elastic characteristics of the crystal. The measured quantities of these constants provide useful information about the toughness and stability of a chemical. By determining the parts of the stress tensor for minute distortion and applying energy in accordance with the lattice contortion that preserves the volume, the elastic factors of the composites were computed at nil pressure [21]. The IRelast program included in Wien2k, which is tailored exactly for cubic systems, was used to determine the elastic numbers. The three elasticity numbers were C_11_, C_12_, and C_44_ due to the cubic crystal lattice symmetry. Table 2 lists these independent constants in summary form. The subsequent requirements of the flexible factors must be met in order for the cubic crystal structure to be mechanically stable: C_11_ − C_12_ > 0; B > 0; C_11_ > 0; C_44_ > 0; C_11_ + 2 C_12_ > 0 [22]. Here, the elastic constants were measured. The elastic stability of our compounds is revealed by their Cij values. AgSbF_3_ has a C_11_ of 46.02 GPa, which is lower than AgBiF_3_ value of 69.74 GPa. AgBiF_3_ is therefore harder than AgSbF_3_. Elastic anisotropy, or crystal A, which is particularly applicable in engineering studies, is closely associated with a material’s capacity to develop minute cracks. We determined the A (anisotropy feature) to compute the flexible anisotropy of these ingredients as follows from the numbers supplied for these elastic factors:

A = 2 C_44_/(C_11_ − C_12_) (1)

Any amount greater or less than 1 denotes anisotropy, whereas A = 1 for an isotropic material. Both of these resources are anisotropic since their measured quantities of A deviate from 1, and the degree of variance reveals the composite anisotropic behavior, as can be seen from the computed data in Table 2.

As the values are −0.369 for AgSbF_3_ and 8.391 for AgBiF_3_, respectively, it is shown that both compounds exhibit a sizable amount of anisotropy. The following formulas must be used to apply elastic constants to obtain the shear modulus G, Young’s modulus E, and Poisson’s ratio v [23,24,25]:

(2) $$E=\frac{9\mathrm{BG}}{G+3B}$$

(3) $$v=\frac{3B-2G}{2\left(G+2B\right)}$$

(4) $$G_{v}=\frac{C_{11}-C_{12}+3C_{44}}{5}$$

(5) $$G_{R}=\frac{5C_{44}\left(C_{11}-C_{12}\right)}{4C_{44}+3C_{11}-C_{12}}$$

(6) $$A=\frac{2C_{44}}{C_{11}-C_{12}}$$

These equations can be used to calculate the quantities of E, A, v, and G, which are displayed in Table 2. A variety of parameters can be used to assess a material’s ductility or brittleness. The change in C_11_ and C_44_ represents Cauchy’s pressure and the type of ductility [26]. The material exhibits ductility if the change in C_11_ and C_44_ is positive, and it exhibits brittleness if the difference between the two is negative. Both materials have positive Cauchy’s pressures, which are 7.064 GPa for AgSbF_3_ and 58.64 GPa for AgBiF_3_, showing that both materials exhibit ductile properties. Another method to determine if something exhibits brittle behavior or ductility is to use the Pugh ratio, or B/G. If a compound with a large Pugh ratio is assumed to be of extraordinary ductility, the peak worth of the B/G is 1.75 [27]. In the present case, the limiting points for the individual complexes are 10.065 for AgSbF_3_ and 13.055 for AgBiF_3_. AgBiF_3_ has more ductility than AgSbF_3_ as a result. To distinguish the ductility and brittleness characteristics of materials, T. Frantsevich et al. [28,29,30] used v (Poisson’s ratio) and described a key value of 0.26. Materials that are brittle have v values that are lower than 0.26, and materials that are ductile have a v values that are higher than 0.26. Both ternary AgZF_3_ (Z = Sb, Bi) compounds, as shown in Table 2, exhibit v values greater than 0.26, namely, 0.528 for AgSbF_3_ and 0.447 for AgBiF_3_, evidencing their ductility. Regarding AgZF_3_ (Z = Sb, Bi), our findings demonstrate that the composites are strong, anisotropic, mechanically ductile, and crack-resistant. Based on these results, we can definitely envision applications for their elastic properties in a range of contemporary electronic technologies.

## 3. Optical Properties

The materials were subjected to energetic incident photons with energies ranging between zero and 14.0 eV on up, and we estimated the optical properties of both complexes using the predicted lattice numbers in balance situations. The dielectric function ε(ω) can be used to determine all optical parameters ε(ω).

### 3.1. The Refractive Index

**Figure 5:**
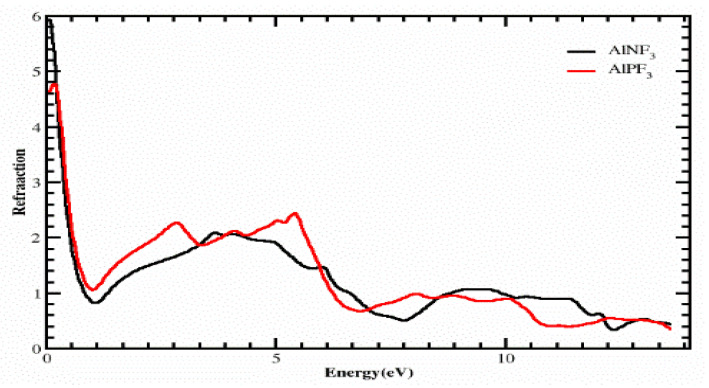
Calculated refractive indexes of compounds AgZF_3_ (Z = Sb, Bi).

**Figure 6:**
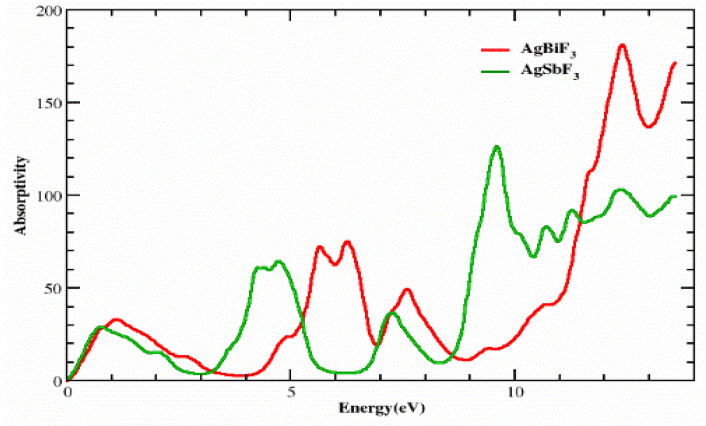
Computed absorption coefficients of complexes AlRF_3_ (R = N, P).

The formulas ε_1_(ω) and ε_2_(ω) were used to calculate a number of physical qualities, such as the refractive index η(ω), optical conductivity σ(ω), absorption coefficient I(ω), and reflectivity R(ω). Figure 5 displays the estimated η(ω). The static refractive index, η(0), is at 0 eV and has values of 7.50 and 9.0 for AgBiF_3_ and AgSbF_3_, respectively, according to the refractive index spectrum. The graph of η(ω) for the composites does not match with a slight adjustment, as can be seen in Figure 5. According to Figure 5, AgBiF_3_ has other refractive index peaks of 2.30 and 2.10 at photon energies of 5.50 and 11.50, respectively, while for AgSbF_3_, other refractive index peaks are observed: 2.30 and 2.30 for energies of 4.0 and 6.0 eV, respectively. We can determine how much light the compound has refracted, and specifically if it may be employed in photoelectric applications, by looking at the refractive index value. From Figure 6, it is evident that photons encountered obstacles when they entered the complex as a result of their interaction with electrons; this explains why there are refractive indexes of more than one (η(ω) > 1).

The more photons are redirected as they pass through a substance, the larger the refractive index of that substance AgSbF_3_ demonstrates more refraction than AgBiF_3._ Every process that increases a material’s electron density also advances its refractive index.

### 3.2. The Absorption Coefficient

When the dielectric function is used, as shown in Figure 6, the absorption factor I(ω) can be identified. According to Figure 6, the chosen compounds have significant absorption factors at energies between 8.5 eV and 14.0 eV for both AgSbF_3_ and AgBiF3. AgSbF_3_ and AgBiF_3_ each have the same threshold values, which are 0 and 0 at 0 eV, respectively. AgSbF_3_ exhibits distinct absorption peaks at energies of 0.8, 4.2, 4.9, 9.5, and 12.5 eV with values of 30, 65, 70, 140, and 100, respectively. In contrast, AgBiF_3_ exhibits absorption peaks of 35, 80, 82, 50, and 195 at energies of 1.2, 5.5, 6.5, 7.6, and 12.5 eV, respectively. At low energies, the compound AgSbF_3_ exhibits a low level of absorption and is comparatively transparent. These substances have strong levels of absorption at high energy levels.

### 3.3. The Reflectivity

**Figure 7:**
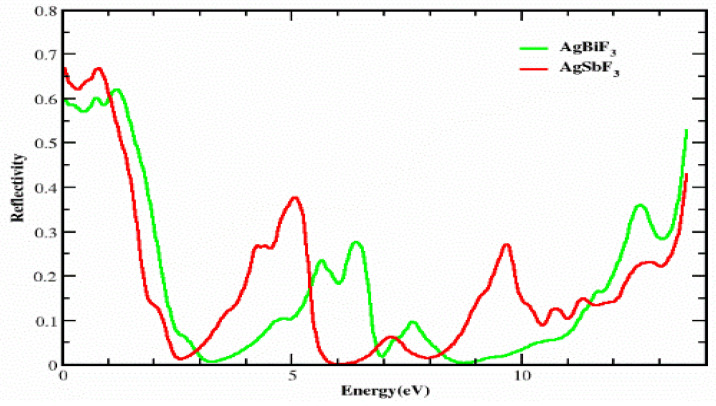
The reflectivity R(ω) of light from the composites AgZF_3_ (Z = Sb, Bi).

Figure 7 displays the R(ω) vs. energy, which was calculated based on the dielectric permittivity, over the energy array of 0.0 eV to 14.0 eV. The reflectance R(0) values at zero frequency for AgSbF_3_ and AgBiF_3_ are 0.6 and 0.66, respectively. For both compounds, it remains approximately in the same range, up to 1.5 eV. The reflectance of AgSbF_3_ first drops to 0.01 at 2.5 eV, then climbs to a peak value of 0.4 at 5.0 eV, then drops to 0.0 at 6.0 eV, and so on: as the photon energy increases, its reflectivity surges. The AgBiF_3_ compound has distinct peaks at 5.5, 6.5, and 12.6 eV, with values of 0.25, 0.31, and 0.35, respectively. As the photon energy increases, the reflectivity of AgBiF_3_ increases as well. AgSbF_3_ is more transparent than AgBiF_3_ in the energy ranges of 0 to 1.5 eV, 3 to 5.5 eV, and 8.2 to 12.5 eV because AgSbF_3_ has a much lower reflectance than AgBiF_3_ in these ranges. The transparency of the materials suggests that these complexes can be employed to create lenses.

### 3.4. Optical Conductivity

**Figure 8:**
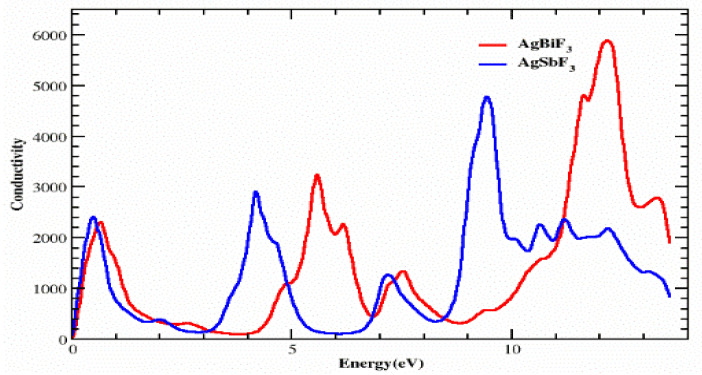
Figured conductivity of incident photon through AgZF_3_ (Z = Sb, Bi) compounds.

The electron movement within a material, brought on by the use of an electromagnetic field, is described by the mathematical symbol σ(ω), which is used to indicate photon conductivity. We can explore the conductivity σ(ω), which is shown in Figure 8, using the dielectric function. For both the compounds, the photon conductivity starts at 0 eV and increases to a value of 2400 at 5.6 eV before falling to 150 in the energy range of roughly 1.0 to 3.3 eV. Then, for the compound AgSbF_3_, we observed the following peaks: 3000, 1500, and 4900 at 4.2, 7.3, and 9.5 eV, respectively. However, for AgBiF_3_, the peaks are 3300, 1400, and 5900 at 5.9, 7.5, and 12.4 eV, respectively. We discovered that in comparison to AgSbF_3_, the compound AgBiF_3_ is visually more conductive at a higher energy, while in the energy range of 8.5 to 11.2 eV, AgSbF_3_ has more conductivity.

## 4. Computational Methodology

The Wien2K simulation engine [31] incorporates this computation, which was carried out using the FP-LAPW method, for the compounds [32,33,34,35,36,37,38,39,40,41,42]. Using the TB-mBJ technique, the electronic characteristics and other characteristics, including the optical characteristics and the density of states, were calculated [36]. With the TB-MBJ approximation, the exchange-correlation potential for a construction and its flexible characteristics are controlled [37]. This work investigates a variety of FP-LAPW base utilities up to RMT, which represents the least radii in muffin-tin spheres and is equivalent to 8.0, as well as Kmax in our work, in which Kmax in the growth of a constant phase wave that has the positive value of the maximum k, in order to achieve a significant degree of convergence. The RMT radii in muffin-tin spheres for the AgZF_3_ (Z = Sb, Bi) compounds and F are 2.00, 1.87, and 2.5 atomic units (au). The Fourier expansion charge density was decreased to Gmax = 13, while the sp herical harmonics were increased within the muffin-tin spheres to lmax = 11(au). The self-consistent field computations are said to meet when the whole energy falls inside the energy range 0.001Ry. By comparing the curves of energy and volume to the electronic position equation put forth by Birch and Murnaghan [38], we were able to obtain the physical parameters. IRelast was used to calculate the flexible numbers for the cubic crystal equilibria, and these data were then applied to analyze the elastic details [41,42].

## 5. Conclusions

In this research study, which was submitted, we fruitfully investigated the structural, optical, electrical, and elastic aspects of ternary fluoro-perovskite materials, AgZF_3_ (Z = Sb, Bi). These are the most accurate and original results. We came to the conclusion that the AgZF_3_ (Z = Sb, Bi) materials are cubically and structurally stable on the basis of their ideal structural parameters. Using the IRelast program, it was possible to forecast elastic parameters such as Young’s modulus, the Pugh ratio, anisotropy factor, Poison’s ratio, ductility, and the Cauchy pressure. These findings reassure us that these materials can be applied to a wide range of modern electrical systems. Using the TB-MBJ potential approximation method, we examined at the fundamental electrical characteristics of the relevant chemicals. We found that both the compounds are metal, and both have an indirect band gap with symmetry points (X-M). The primary sources of the valence band in the DOS are the F-tot, F-p, and Ag-d states, which are all in the valence band for AgBiF_3_, have energies ranging from −4.02 to −6.40 eV and from 0 to −2.50 eV, and make prominent contributions. Bi-p, F-tot, and Bi-tot make small contributions in the conduction band in the energy range from 0.0 to 1.00 eV for the same compound. For AgSbF_3_, F-tot, Ag-tot, F-p, and Ag-d make the largest contributions in the valance band in the energy ranges from −3.20 to −5.40 eV, from −3.80 to −5.40 eV, from −3.20 to −5.40 eV, and from −3.80 to −5.40 eV, respectively. Similarly, in the conduction band for the same compound, the major contribution comes from Sb-p, whereas the F-tot in the energy range from 0 to 1.00 eV for both the states is as illustrated in Figure 4.

## Figures and Tables

**Table 1:** Optimized structural characteristics of AgZF_3_ (Z = Sb, Bi) consequent to using the Birch–Murnaghan state vs. volume.

Compounds	a_o_ (Å)	B (GPa)	B’	V_0_ (a.u^3^)	E_0_ (Ry)
AgSbF_3_	4.42	66.38	5.42	583.14	−24,201.03
AgBiF_3_	4.45	65.34	5.08	594.10	−54,396.93

**Table 2:** The designed elastic numbers, bulk modulus, anisotropy factor, Young’s modulus, Poison’s ratio, Pugh ratio (B/G), and Cauchy’s pressure for ternary AgZF_3_ (Z = Sb, Bi) complexes.

Compounds	AgSbF_3_	AgBiF_3_
**C_11_**	14.61	69.74
**C_12_**	46.02	67.09
**C_44_**	7.64	11.12
**G**	6.60	5.01
**A**	−0.37	8.39
**v**	0.528	0.45
**B/G**	10.07	13.06

## Data Availability

Not applicable.

## Sample Availability

Samples of the compounds are available from the authors.
